# Molecular characterization of clinical multidrug-resistant *Klebsiella pneumoniae* isolates

**DOI:** 10.1186/1476-0711-13-16

**Published:** 2014-05-01

**Authors:** Xiaoli Cao, Xuejing Xu, Zhifeng Zhang, Han Shen, Junhao Chen, Kui Zhang

**Affiliations:** 1 Department of Laboratory Medicine, Nanjing Drum Tower Hospital, the affiliated Hospital of Nanjing University Medical School, Zhongshan Road, 321#, Gulou District, Nanjing, Jiangsu Province 210008, PR China

**Keywords:** Multidrug resistance, Resistance determinants, Multi-locus sequence typing, Pulsed-field gel electrophoresis, Plasmid replicons

## Abstract

**Background:**

*Klebsiella pneumoniae* is a frequent nosocomial pathogen, with the multidrug-resistant (MDR) *K. pneumoniae* being a major public health concern, frequently causing difficult-to-treat infections worldwide. The aim of this study was to investigate the molecular characterization of clinical MDR *Klebsiella pneumoniae* isolates.

**Methods:**

A total of 27 non-duplicate MDR *K. pneumoniae* isolates with a CTX-CIP-AK resistance pattern were investigated for the prevalence of antimicrobial resistance genes including extended spectrum β-lactamase genes (ESBLs), plasmid-mediated quinolone resistance (PMQR) genes, 16S rRNA methylase (16S-RMTase) genes, and integrons by polymerase chain reaction (PCR) amplification and DNA sequencing. Plasmid replicons were typed by PCR-based replicon typing (PBRT). Multi-locus sequence typing (MLST) and pulsed-field gel electrophoresis (PFGE) were carried out to characterize the strain relatedness.

**Results:**

All the isolates co-harbored 3 or more resistance determinants. OqxAB, CTX-M-type ESBLs and RmtB were the most frequent determinants, distributed among19 (70.4%),18 (66.7%) and 8 (29.6%) strains. Fourteen isolates harbored class 1 integrons, with *orfD*-*aacA4* being the most frequent gene cassette array. Class 3 integrons were less frequently identified and contained the gene cassette array of *bla*_GES-1_-*bla*_OXA-10_-*aac(6′)-Ib*. IncFII replicon was most commonly found in this collection. One cluster was observed with ≥80% similarity among profiles obtained by PFGE, and one sequence type (ST) by MLST, namely ST11, was observed in the cluster.

**Conclusion:**

*K. pneumoniae* carbapenemase (KPC)–producing ST11 was the main clone detected. Of particular concern was the high prevalence of multiple resistance determinants, classs I integrons and IncFII plasmid replicon among these MDR strains, which provide advantages for the rapid development of MDR strains.

## Background

*Klebsiella pneumoniae* is an opportunistic pathogen associated with both community-acquired and nosocomial infections, including pneumonia, urinary tract infections, septicemia and wound infections, with the increasingly multidrug-resistant (MDR) *K. pneumoniae* being a major public health concern.

The prevailing hypothesis is that these bacteria acquire multidrug resistance through horizontal transfer of antimicrobial resistance genes mediated by mobile genetic elements such as integrons [1]. Several genes that are frequently involved in multidrug resistance to commonly used antimicrobial agents include plasmid-mediated quinolone resistance (PMQR) genes [2], exogenously acquired 16S rRNA methyltransferase (16S-RMTase) genes [3], and extended-spectrum β-lactamases (ESBLs) encoding genes [4]. Furthermore, carbapenem-hydrolyzing β-lactamases (CHβLs) has rapidly emerged in recent years, with *K. pneumoniae* being the most common organism associated with *K. pneumoniae* carbapenemase (KPC) resistance determinants [5].

Although the high prevalence of these resistance determinants has been reported among MDR *K. pneumoniae* strains [6,7], there is little information available on the distribution of integrons and plasmid replicons among these strains, and limited data on the genetic relationship between these strains.

The aim of this study therefore was to investigate the prevalence of frequently reported antimicrobial resistance determinants among the 27 clinical MDR *K. pneumoniae* isolates, to explore the distribution of integrons and plasmid replicons, as well as to analyze the genetic clonality by pulse field gel electrophoresis (PFGE) and multi-locus sequence typing (MLST).

## Method

### Bacterial isolates

A total of 27 non-duplicate MDR *K. pneumoniae* isolates simultaneously displaying resistance to cefotaxime, amikacin and levofloxacin were collected from our hospitals from March to July 2011(Susceptibility to antimicrobial agents was determined using Kirby–Bauer’s disc diffusion method and the results were analyzed and interpreted according to CLSI guidelines [8]). Among them, 12 strains were resistant to imipenem. The clinical sources of these specimens include sputum (n = 13), urine (n = 4), blood (n = 7), and wound secretions (n = 3). Among these 27 isolates, 2 strains originated from community-acquired infections, defined as the pathogen being isolated within 48 h after hospital admittance. The other 25 ones were from nosocomial infections, where the pathogens were isolated more than 48 h after being admitted to a hospital.

### Detection of antimicrobial resistance determinants

DNA templates were prepared by the boiling method. All the isolates were analyzed for the presence of *bla*ESBLs (*bla*CTX, *bla*TEM, *bla*SHV, *bla*VEB, and *bla*OXA) [9], PMQRs (*qnrA*, *qnrB*, *qnrC*, *qnrD*, *qnrS*, *aac (6′)-Ib-cr*, and *qepA*) [10], and 16S-RMTases (*armA*, *npmA*, *rmtA*, *rmtB*, *rmtC*, *rmtD*, and *rmtE*) [11]. The 12 strains resistant to imipenem were further analyzed for CHβLs endocing genes (*bla*KPC, *bla*OXA-48, *bla*IMP, *bla*VIM, *bla*NDM, *bla*DIM, *bla*SPM, and *bla*SIM) [12]. All genes were screened by multiplex PCR followed by single PCR for gene confirmation. Positive products were further purified with a DNA purification kit and then sent to the Majorbio Company (Shanghai, China) for sequencing. Sequences were analyzed by using the Chromas-Pro application and BLAST (http://www.ncbi.nlm.nih.gov/BLAST).

### Detection of integrons

Integrons were detected by PCR amplification of class 1, 2, and 3 integrase-specific *int-1*, *int-2*, and *int-3* genes, as described previously [13]. To reveal the gene cassettes of the variable region of integrons, the variable region of class 1 and 3 integrons were further amplified and purified, and then sequenced to determine their gene cassette composition. The resulting nucleotide sequences and deduced protein sequences were analyzed with the BLAST and FASTA programs of the National Center for Biotechnology Information (http://www.ncbi.nlm.nhi.gov).

### PCR-based replicon typing

Main plasmid incompatibility groups including F, FIA, FIB, FIC, HI1, HI2, I1-Ic, L/M, N, P, W, T, A/C, K, B/O, X, Y, and FII were determined using the PCR-based replicon typing (PBRT) scheme as described by Carattoli [14].

### Pulsed-field gel electrophoresis

Clonal relatedness of 27 *K. pneumoniae* isolates was analyzed by pulsed-field electrophoresis (PFGE) as described [15]. Prepared genomic DNA was digested using the restriction endonuclease *Xba*I (Fermentas, ABI, Germany), and DNA fragments were separated in a PFGE CHEF-DR III system (Bio-Rad Laboratories, Hercules, CA) in 0.5× Tris-borate-EDTA buffer at 120 V for 19 h, with pulse times ranging from 2.2 s to 54.2 s. The banding patterns were analyzed by the BioNumerics software (Applied Math, Sint-Maten-Latem, Belgium). Cutoff lines at 65% and 80% were used to analyze genetic relatedness.

### Multi-locus sequence typing

Genotyping for the 27 MDR strains was further determined by multi-locus sequence typing (MLST) analysis. MLST with 7 genes (*gapA*, *infB*, *mdh*, *pgi*, *phoE*, *rpoB*, and *tonB*) was performed according to Diancourt et al. [16]. Alleles and sequence types (STs) were assigned by using the MLST database (http://www.pasteur.fr/mlst/Kpneumoniae.html).

## Results

### Wide distribution of resistance determinants

**Figure 1:**
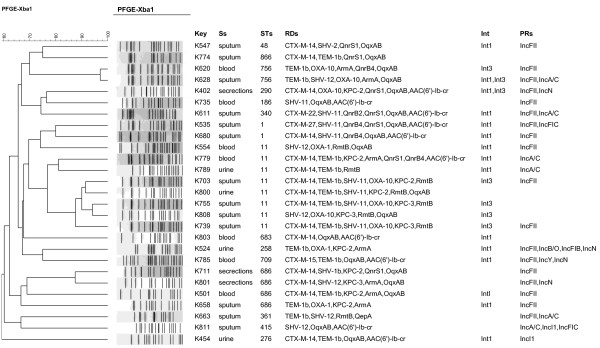
**Dendrogram based on pulse-field gel electrophoresis developed in BioNumerics for 27 clinically multidrug-resistant *Klebsiella pneumoniae *isolates**. Ss, specimens; Int, integron; STs, sequence types; RTs, resistance determinants; RRs, plasmid replicons.

Diverse resistance determinants were found among the MDR strains (Figure 1). β-Lactamse including CTX-M-type ESBLs, sulfhydryl variable (SHV) variants, TEM-1b, KPC, and OXA, were carried by 18 (66.7%), 15 (55.6%), 13 (48.1%), 15 (55.6%), 12 (44.4%), and 10 (37.0%) of the isolates, respectively. PMQRs were detected in 21 isolates. Of these, 19 (70.4%) belonged to OqxAB, 11 (40.7%) to Qnr (7 QnrS1, 1 QnrB2, and 4 QnrB4), 10 (37.0%) to AAC (6′)-Ib-cr, and 1 to QepA. Determinants conferring resistance to amikacin included 15 (55.6%) 16S-RMTase determinants, with RmtB being present in 29.6% (8/27), ArmA in 25.9% (7/27), AAC(6′)-Ib-cr in 10 (37.0%), and AAC(6′)-Ib in 10 (37.0%) (encoded by 10 *aacA4* located within class 1 integron).

Furthermore, all the isolates co-harbored 3 or more resistance determinants. The PMQRs were present in 19 out of the 22 ESBL-carrying *K. pneumoniae* isolates. Of the 11 KPC-positive MDR *K. pneumoniae* isolates, 7 co-carried *rmtB* or *armA*.

### Prevalence of integrons

Integron analysis showed that 14 isolates carried class 1 integrons; 6 carried class 3 integrons; and 2 contained both of class 1 and 3 integrons (Figure 1). No class 2 integrons were found. Sequencing analysis for intI1-positive strains revealed that the cassette arrays of class 1 integron include *orfD*-*aacA4* (n = 10 strains) and *aad5*-*dfrA17* (n = 4 strains). Moreover, the variable regions in class 3 integrons of all the 6 isolates were the same as those comprising *bla*_GES_-1-*bla*_OXA-10_-*aac(6′)-Ib*, which has been identified in an earlier study [17].

### Distribution of plasmid replicons

PBRT revealed that IncFII type was the predominant plasmid replicon among the MDR *K. pneumoniae* strains (Figure 1), IncFII (18/27, 66.7%), IncA/C (6/27, 22.2%) and IncI1 (2/27, 7.4%) were found alone or in combination; IncN(4/27, 14.8%), IncFIC (2/27, 7.4%), IncY(1/27, 3.7%), IncFIB (1/27, 3.7%) and IncB/O (1/27, 3.7%) were found in combination with IncFII.

### Clonal relatedness

According to the PFGE patterns of the isolates (Figure 1), 27 different clonal patterns were observed among the 27 MDR *K. pneumoniae* isolates with ≥65% similarity.

One cluster of 7 closely related isolates was found that exhibited ≥80% similarities, these isolates were identified to be ST11 clones.

### Sequence types for the *K. pneumoniae* isolates

MLST was conducted to determine the extent of genotypic diversity among the *K. pneumoniae* isolates. Fifteen different STs were identified. The most dominant ST was ST11 (29.6%, 8/27), followed by ST686 (14.8%, 4/27), ST1 (7.4%, 2/27), ST756 (7.4%, 2/27). These 4 STs accounted for 59.3% (16/27) of the total isolates. Among the STs identified, ST415, ST186, ST276, and ST866 have previously not been identified to be MDR strains.

## Discussion

MDR *K. pneumoniae* strains have caused major therapeutic problems worldwide. The increasing prevalence of clinical MDR isolates has been associated with higher morbidity and mortality rates, posing a considerable threat to public health. In this study, we provide the current data on the molecular characterization of MDR *K. pneumoniae* isolates isolated from different clinical samples of hospitalized patients.

Our study revealed a wide distribution of diverse resistance determinants among the MDR *K. pneumoniae* strains. The most prevalent determinants were *oqxAB* genes, which have been reported to be the most frequent PMQRs in *K. pneumoniae* isolates [18]. CTX-M has been found to be widely disseminated among clinical *Enterobacteriaceae* such as *Escherichia coli* and *K. pneumoniae*[19], the even higher prevalence of *bla*CTX-M among our strains corresponds with an earlier report showing that CTX-M–producing strains are generally MDR ones [19]. Furthermore, in line with the previous investigation [20], KPC is highly prevalent among our strains, which also demonstrated that the production of KPC-type carbapenemases was the most prevalent carbapenem resistance mechanism in *K. pneumoniae* isolates. In addition, the widely distributed 16S-RMTase encoding genes in our MDR strains have been previously reported in China [21], together with AAC (6′)-Ib-cr and AAC (6′)-Ib accounting for the amikacin resistance. Recently, co-production of 16S rRNA methylases (ArmA and RmtB) and KPC were frequently reported in *Enterobacteriaceae*[22], which was also observed in our study, leading to few choices for antimicrobial treatment. Notably, there seems to be an intimate association between the occurrence of *oqxAB* and ESBLs; a similar phenomenon was noted for 16S-RMTase and β-lactamase. Such a frequent co-existence of 16S-RMTase with PMQRs and β-lactamase among MDR *K. pneumoniae* isolates in our study suggests a horizontal dissemination of these determinants amongst clinical MDR *K. pneumoniae* isolates. These MDR strains co-carrying diverse and numerous multiple resistance determinants may impose limitations in the therapeutic options available for the treatment of infections.

Epidemic resistance plasmids including IncFII, IncA/C, IncL/M, IncN and IncI1 plasmids has been worldwide detected in *Enterobacteriaceae* of different origin and sources [23]. In our study, the wide distribution of IncFII plasmid replicon is in accordance with the previous studies showing that IncFII plasmid replicons were most frequently detected in CTX-M-producing *enterobacterial* isolates in China and in Europe [24,25]. Furthermore, other plasmid replicons such as IncN, IncI1, IncY and IncA/C distributed among our strains have also been previously reported [26,27]. Additionally, the widely prevalent IncFII plasmid replicons and class I integrons identified in our study indicate that they might be playing an important role in attributing MDR to the clinical *K. pneumoniae* isolates. It is noteworthy that class 3 integrons were also detected among our strains. which is also reported earlier by Qi et al. in 2011 [28], showing the presence of class 3 integrons in MDR *K. pneumoniae* isolates from clinical settings in China, since class 3 integrons have also been found and characterized in Europe [29].

The genetic diversity of our isolates revealed that most isolates were of different strains, indicating the ease of transmission of these resistance determinants between bacterial species by mobile elements. This has been partially confirmed by the high prevalence of integrons and plasmid replicons in our study. However, it seems that the epidemic dissemination of the major clone ST11 producing *bla*KPC also played an additional role in our study, this corresponds with the report that ST11 is a truly international sequence type and associated with KPC [30]. Which is partly different from the KPC-producing *K. pneumoniae* clonal complex 258 (ST258, ST512, and ST101) in Europe and USA [31,32]. Of note, the other STs such as ST415, ST186, ST276, and ST866 have not been previously identified to be MDR clones. Such a high heterogeneity of ST clones observed may further indicate the role of plasmids and integrons in the development of clinical MDR isolates.

## Conclusion

In summary, our study showed that clinical MDR *K. pneumoniae* isolates may result mainly from the horizontal dissemination of multiple resistance determinants and the clonal dissemination of MDR ST11 strains–producing KPC-2, which alerts us the emergency and necessity to vigorously implement the infection control practice to prevent the dissemination of these MDR isolates in the healthcare settings.

## Abbreviations

MDR: Multidrug-resistant; MLST: Multi-locus sequence typing; PFGE: Pulsed-field gel electrophoresis; KPC: *K. pneumoniae* carbapenemase; PMQRs: Plasmid-mediated quinolone resistance genes; 16S-RMTase: 16S rRNA methyltransferase; CHβL: Carbapenem-hydrolyzing beta-lactamase; ESBL: Extended spectrum β-lactamase; ST: Sequence type; PBRT: PCR-based replicon typing.

## Competing interests

The authors declare that they have no competing interests.

## Authors’ contributions

XC performed experimental work and drafted manuscript; XX and ZZ analyzed the study data; JC and HS provided interpretation of data; KZ conceived the study and provided data interpretation. All authors read and approved the final manuscript.
